# Supplementary material for: The Optimal Concentration of Formaldehyde is Key to Stabilizing the Pre-Fusion Conformation of Respiratory Syncytial Virus Fusion Protein
Source: Viruses. 2019 Jul 8;11(7):628. doi: 10.3390/v11070628 (PMC6669674; doi:10.3390/v11070628)
Supplement: Supplementary file 1 [file viruses-11-00628-s001.pdf]

Fig. S1

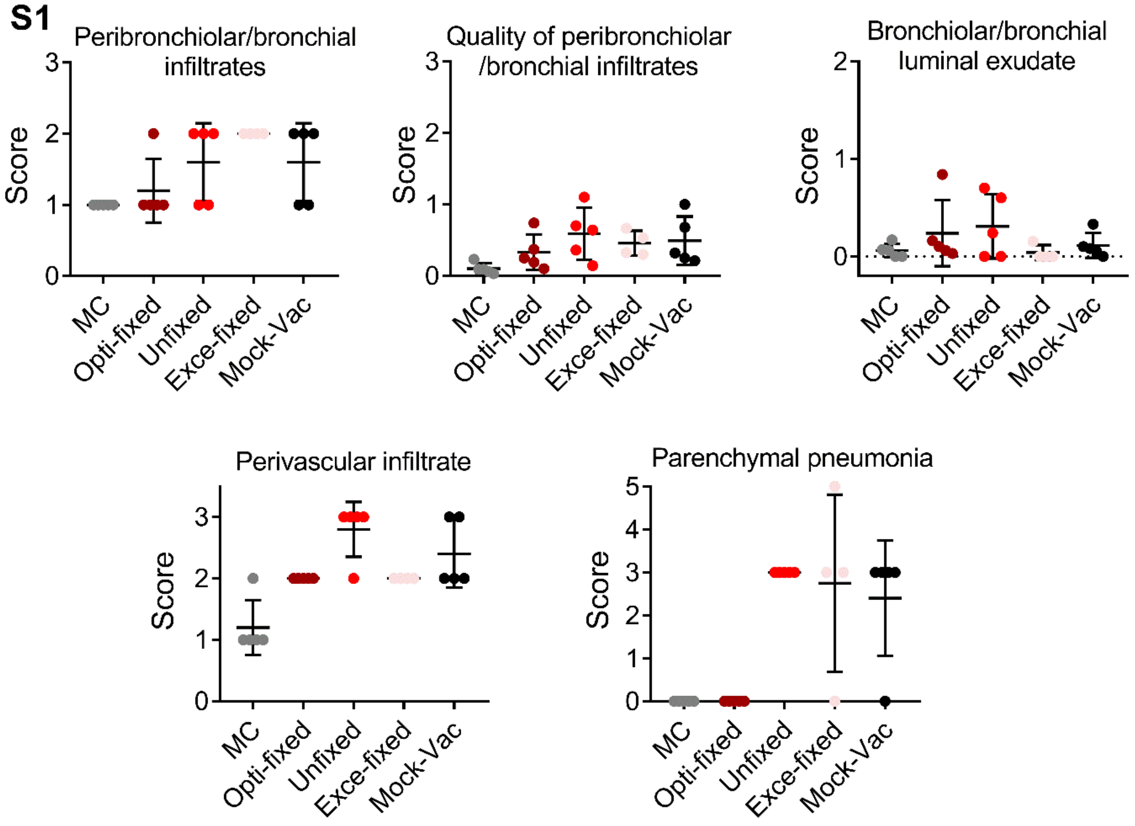

Figure S1. Pathological evaluation of inoculated mice after RSV A2 challenge. The left lungs of five mice from each group were obtained on day 5 post-challenge and stored in 10% formaldehyde solution prior to being paraffin-embedded and sectioned. After stained, several pathological indexes of these sections, including peribronchiolar/bronchial infiltrates, the quality of peribronchiolar/bronchial infiltrates, bronchiolar/bronchial luminal exudate, and perivascular infiltrate and parenchymal pneumonia, were evaluated to calculate the total pathology score. MC means mock challenge; opti-fixed means mice inoculated with 0.0244% formaldehyde-treated infected cells (optimal concentration); unfixed means mice inoculated with PBS-treated infected cells; exce-fixed means mice inoculated with 25% formaldehyde-treated infected cells (excessive fixative); mock-vac means mice inoculated with an equal quantity of uninfected cells.

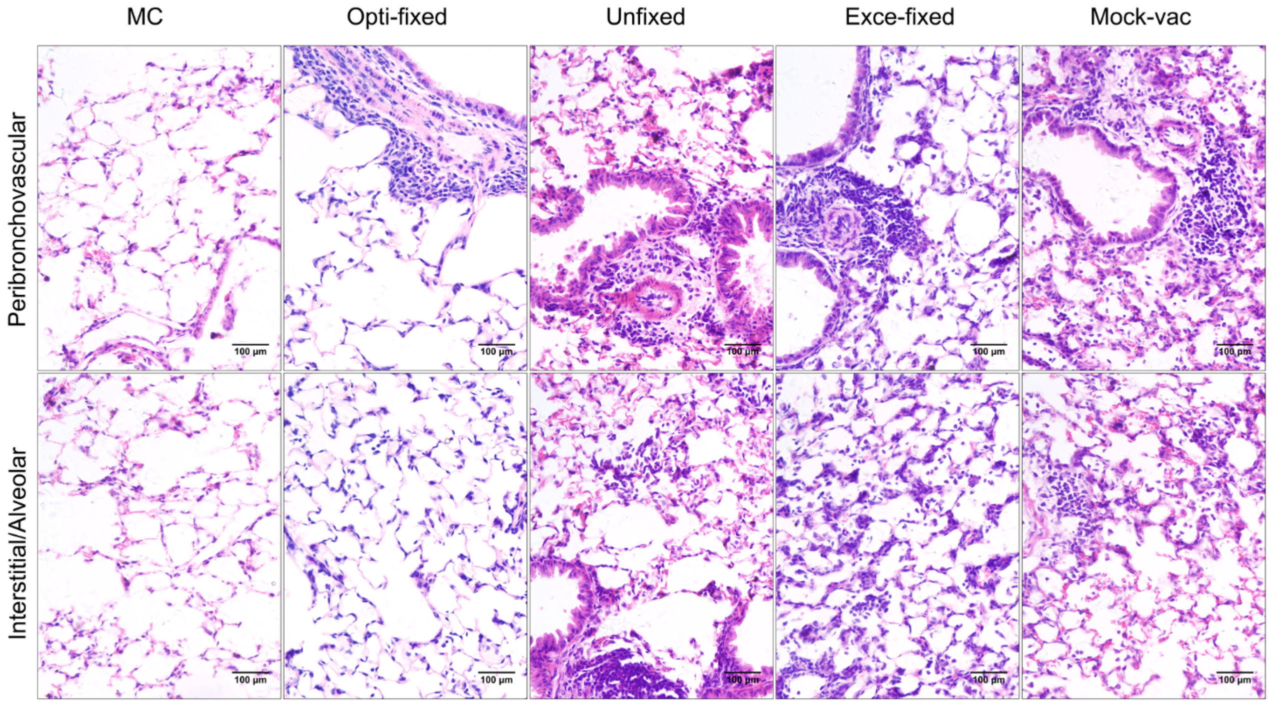

Figure S2. H&E staining of pulmonary pathological sections. The left lungs of five mice from each group were obtained on day 5 post-challenge and stored in 10% formaldehyde solution prior to being paraffin-embedded and sectioned. Scales bar for H&E staining indicate 100 μm. Refer to Figure S1 for the meaning of each group name.

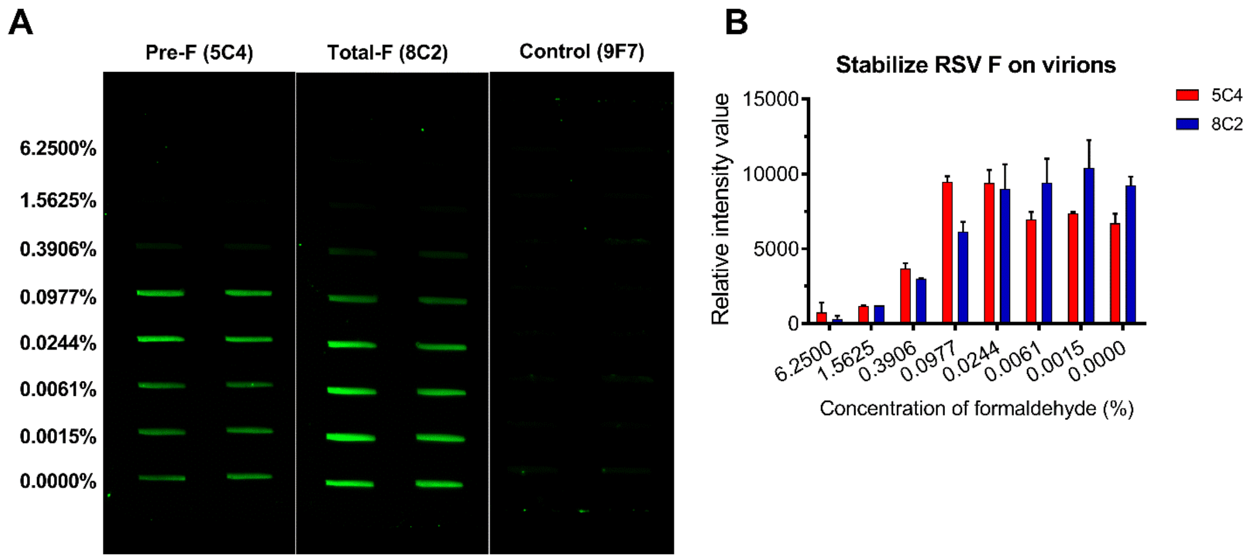

Figure S3. The optimal formaldehyde concentration is beneficial to the stability of pre-F protein on virions. RSV A2 was treated with PBS gradient diluted formaldehyde solution and incubated at room temperature for 12 hours.The treated virus was then dialyzed into a PBS buffer and kept there for 16 hours. Finally, the F protein in the sample was detected by Dot-Blot (A), and we showed here a representative result from three independent replicates. (B) The quantization of signals in Dot-Blot represents the data of three independent experiments, which means geometric mean ± 95% CI.
